# Editorial: Microbial Control of the Nitrogen Cycle

**DOI:** 10.3389/fmicb.2020.00950

**Published:** 2020-05-14

**Authors:** Juan Sanjuan, María J. Delgado, Lourdes Girard

**Affiliations:** ^1^Departamento de Microbiología del Suelo y Sistemas Simbióticos, Estación Experimental del Zaidín, CSIC, Granada, Spain; ^2^Programa de Biología de Sistemas y Biología Sintética, Centro de Ciencias Genómicas, UNAM, Cuernavaca, Mexico

**Keywords:** nitrogen oxides, environmental pollution, climate change, sustainable food production, nitrogen fixation

Plant biomass and productivity of many ecosystems are limited by the availability of reactive nitrogen, this is reduced or oxidized forms of N. Over the last century synthetic N fertilizers have undoubtedly helped to enhance crop yields, thus allowing an unprecedented growth of the world population. Despite this, nearly 80% of N fertilizer applied to crops does not reach human mouths but is lost to the environment in water run-off from fields, animal waste, and gas emissions. This N fertilizer abuse, particularly in rich countries, has strongly perturbed the biogeochemical N cycle with an unprecedented production and accumulation of reactive N in the biosphere that threatens human health and ecosystems biodiversity, and contributes to climate change. Optimizing N use and minimizing its negative impacts is a major challenge for the next decades (Galloway et al., 2013).

Microbes (bacteria and fungi) are main protagonists of all the major reactions in the N-cycle. Microbial activities that process nitrogen in soils and sediments are complex and highly challenging. Better understanding of these processes is important for improving the efficiency of agricultural practices that are essential for food production and environmental quality across the globe. Therefore, any technologies aiming at reducing the N problem must recognize the roles and potential applications of these N-cycle microbes, from diazotrophs to denitrifiers. These microbiotechnologies are necessary if we want to bring reactive N levels back within planet safety boundaries while ensuring crop productivity and food security (Steffen et al., 2015).

This Research Topic focused on the biotechnology of microbes and microbial processes which can directly or indirectly contribute to alleviate the N problem at local and/or global scales. Fifteen of 20 submitted manuscripts have been accepted for publication.

Five articles dealt with various aspects of nitrogenase and biological nitrogen fixation (BNF), the key bacterial process that converts atmospheric N_2_ into reactive nitrogen accessible for living organisms. Three papers were on the nitrogen fixing symbioses between legume plants and soil rhizobia. Temprano-Vera et al. analyzed differences among *Sinorhizobium fredii* strains at nodulating various genotypes of *Glycine max* (soybean, the most widely produced legume) and *Glycine soja* (the wild ancestor of soybeans). Their results illustrate the complexity of genetic determinants of the soybean-rhizobia compatibility. Also concerning soybeans, Fernández et al. reported the importance of adaptation of the soybean symbiont *Bradyrhizobium japonicum* to microxic conditions for efficient nitrogen fixation, and identified novel genes subjected to posttranscriptional regulation during microxia which are important for symbiosis. Tsyganov et al. reported on the contribution of a legume nitrogen-fixing symbiosis to the plant tolerance to Cd, as well as its potential applications for phytoremediation and phytostabilization of soils with high Cd contents. Two other studies identified novel functions which are important for optimal nitrogen fixation in free-living diazotrophs, and therefore should be considered in current and future studies aiming at obtaining nitrogen-fixing plants by genetic engineering. Navarro-Rodríguez et al. reported on the importance of the molybdenum storage protein MoSto for nitrogen fixation by *Azotobacter vinelandii* under Mo-limiting conditions. On another hand, Nonaka et al. described the essentiality of several proteins required for nitrogenase assembly during diazotrophic growth by the cyanobacterium *Leptolyngbya boryana*.

Another key process in the N cycle is denitrification, which has a strong impact in the environment through the production of reactive nitric oxide (NO) and the powerful greenhouse gas nitrous oxide (N_2_O), with a strong potential to drive climate change. Olaya-Abril et al. have explored this process in *Paracoccus denitrificans* PD1222 through a quantitative proteomic analysis and have established key features of the denitrification metabolism, particularly the induction of enzymes of the TCA and glyoxylate cycles, together with enzymes that synthesize acetyl-CoA. On another hand, Liu B. et al. have monitored the kinetics of denitrification gene expression and NO, N_2_O, and N_2_ production in soils exposed to microxia. They concluded that regulatory strategies observed in individual isolates are also displayed in complex communities, emphasizing the need for successive sampling when identifying active key organisms. Two other manuscripts showed that in addition to denitrification, other microbial processes such as nitrate assimilation can be sources of NO and N_2_O. Ruiz et al. reported that the pathway involving the assimilatory nitrate (NarB) and nitrite (NirB) reductases indirectly contribute to NO synthesis by cooperating with the denitrification pathway in *Sinorhizobium meliloti*. Hidalgo-García et al. demonstrated that in addition to its involvement in nitrate assimilation, NarB is also required for NO and N_2_O production in *Rhizobium etli*. Nitrite produced by NarB from assimilatory nitrate reduction is detoxified by NirK and cNor denitrifying enzymes that convert nitrite into NO which in turn is reduced to N_2_O, respectively.

Nitrification linked to denitrification constitutes an important biotic source of N_2_O emissions from soils. The aerobic oxidation of ammonia (NH_3_) to nitrite (${\text{NO}}_{2}^{-}$) is carried out by ammonia oxidizing bacteria (AOB) or archaea (AOA) that also possess the enzymatic pathway to reduce ${\text{NO}}_{2}^{-}$ to N_2_O (Hu et al., 2015). In this regard, the changes in the microbial communities and N_2_O emissions due to common soil management practices were investigated in various papers. The impact of urea fertilization on the nitrifier microbial populations was evaluated by Staley et al. in eight contrasting agricultural soil types. Their results indicate that while bacterial and archeal diversity is negatively affected, high urea applications favor particular strains or species of nitrifying genera such as *Nitrobacter, Nitrospira*, and *Nitrosospira*, regardless the soil type. In the same line, Lourenço et al. reported that soil amendments with organic vinasses and inorganic N fertilizer lead to increased N_2_O emissions which correlate with enhanced abundance of AOB, particularly *Nitrosospira*. On another hand, Fang et al. reported that application of dazomet, a microbicide used to control soil pathogens, causes reversible changes in bacterial community compositions and reduces the abundance of N-cycle functional genes, however N_2_O production rates increased in correlation with NH_3_, dissolved amino acids and microbial biomass nitrogen contents. Tomasek et al. provided evidence that inundation drives shifts in both the microbial community and the denitrification rates, suggesting that approaches such as periodic flooding can be an effective management strategy to remediate nitrate pollution. Finally, Black and Just reported the influence that river mussels communities have on the abundance of N-cycling microbial species. Mussels filtration and biodeposition determine a sediment habitat that favors the mixotrophic coexistence of nitrite-oxidizing (NOB) and ammonia-oxiding (comammox) *Nitrospira* species, what impacts N-cycling in river backwaters. Another important group of microorganisms that play a significant role in the N cycle are AOA. Liu L. et al. explored a new strategy for the rapid enrichment of high abundance *Nitrosocosmicus*-like AOA from soil, providing a new solution to the enrichment and cultivation of AOA in short periods.

In summary, these articles have brought novel knowledge on the diversity, genetics, and physiology of microbes from terrestrial and aquatic ecosystems that participate in the N cycle. Potential applications of this knowledge can be envisaged that will contribute to alleviate the nitrogen problem to achieve a healthier planet and a sustainable food production.

## Author Contributions

JS initiated the Research Topic and invited editors MD and LG. The editorial was written jointly by the editors of the topic.

## Conflict of Interest

The authors declare that the research was conducted in the absence of any commercial or financial relationships that could be construed as a potential conflict of interest.
